# Neutrophils and Chronic Inflammation

**DOI:** 10.1093/geroni/igaf122.819

**Published:** 2025-12-31

**Authors:** Emily Goldberg

**Affiliations:** University of California, San Francisco, California, United States

## Abstract

Neutrophils are short-lived immune cells that are best known for their important role in antimicrobial immunity. Recently, neutrophils have been implicated in sterile inflammatory diseases like diabetes, cardiovascular disease, and cancer. We have been investigating how neutrophils might contribute to chronic inflammatory diseases, and particularly in age-related inflammation.

